# 125 Community Socioeconomic Status Is Associated with Social Participation Outcomes

**DOI:** 10.1093/jbcr/irac012.127

**Published:** 2022-03-23

**Authors:** Brian M Kelter, Lauren J Shepler, Pengsheng Ni, Lewis E Kazis, Barclay T Stewart, Colleen M Ryan, Jeffrey C Schneider

**Affiliations:** Spaulding Rehabilitation Hospital, Boston, Massachusetts; Spaulding Rehabilitation Hospital, Boston, Massachusetts; Boston University School of Public Health, Boston, Massachusetts; Boston University School of Public Health, Spaulding Rehabilitation Hospital, Harvard Medical School, Boston, Massachusetts; University of Washington, Seattle, Washington; Harvard Medical School, Boston, Massachusetts; , Massachusetts

## Abstract

**Introduction:**

Socioeconomic factors are recognized as important social determinants of health. Data however are sparse describing the relationship between socioeconomic status and long term burn outcomes. This study aims to examine associations between community-level socioeconomic status and social participation outcomes in burn survivors.

**Methods:**

Data was obtained from the Life Impact Burn Recovery Evaluation (LIBRE) Journey study that assesses longitudinal social participation outcomes of community dwelling burn survivors. Subjects were linked to the Distressed Communities Index (DCI), which combines seven indicators into a metric that depicts community economic well-being. Participants were categorized by time since burn (< 5, 5-15, ≥15 years). Linear regression models examined associations between DCI (zip code and county levels) and LIBRE domain scores (Family & Friends, Social Interactions, Social Activities, Work & Employment).

**Results:**

The study included 314 burn survivors, (mean age 44.1 years; 61.0% female; 48.6% married; 82.8% white). The population was distributed among the time since injury categories (< 5: 35.8%, 5-15: 27.5%, ≥15: 36.7%). Approximately 18% of subjects were categorized in the “at risk” or “distressed” DCI categories. For survivors less than five years from burn, a DCI score increase of 1 standard deviation (worse socioeconomic status) at the zip code level was associated with decreased Family & Friends and Social Activity scores of 2.6 (p=.01) and 2.0 points (p=0.04), respectively (small effect sizes). This relationship was even stronger when controlling for sociodemographic factors. In regression analysis, survivors within the first five years from injury living in “at risk” or “distressed” communities showed worse Family & Friend scores by 6.5 points compared to those living in “prosperous” communities, even after adjusting for age, gender, race, ethnicity, education, and marital status (p=0.04; moderate effect size). There were no significant associations between DCI and LIBRE domain scores for survivors assessed beyond 5 years from injury.

**Conclusions:**

Social participation outcomes were worse in burn survivors who lived in socioeconomically disadvantaged neighborhoods. Burn survivors who face socioeconomic challenges may need additional support to address social disparities to improve outcomes.

